# Computational approaches for identifications of altered ion channels in keratoconus

**DOI:** 10.1038/s41433-024-03395-5

**Published:** 2024-10-17

**Authors:** Kiran Bharat Gaikwad, Jayavigneeswari Suresh Babu, K. T. Shreya Parthasarathi, Janakiraman Narayanan, Prema Padmanabhan, Akhilesh Pandey, Seetaramanjaneyulu Gundimeda, Sailaja V. Elchuri, Jyoti Sharma

**Affiliations:** 1 Manipal Academy of Higher Education, Manipal, Karnataka 576104 India; 2 Institute of Bioinformatics, International Technology Park, Bangalore, 560066 India; 3 Department of Nanobiotechnology, Vision Research Foundation, Sankara Nethralaya Campus, Chennai, 600006 India; 4 Department of Cornea, Medical Research Foundation, Sankara Nethralaya, Chennai, 600006 India; 5 Department of Laboratory Medicine and Pathology, Mayo Clinic, Rochester, MN 55905 USA; 6 Center for Individualized Medicine, Mayo Clinic, Rochester, MN 55905 USA

**Keywords:** Corneal diseases, Drug discovery

## Abstract

**Background:**

Keratoconus is an etiologically complex, degenerative corneal disease that eventually leads to loss of corneal integrity. Cells in corneal epithelium and endothelium express various types of ion channels that play important roles in ocular pathology. This emphasizes the need of understanding alterations of ion channels in keratoconus.

**Method:**

Differential gene expression analysis was performed to identify deregulated ion channels in keratoconus patients using transcriptomic data. Thereafter correlation analysis of ion channel expression was performed to obtain the changed correlation between ion channels’ expression in keratoconus patients versus control samples. Moreover, Protein-protein interaction networks and a pathway map was constructed to identify cellular processes altered due to the deregulation of ion channels. Furthermore, drugs interacting with deregulated ion channels were identified.

**Results:**

Total 75 ion channels were found to be deregulated in keratoconus, of which 12 were upregulated and 63 were downregulated. Correlations between ion channel expressions found to be different in control and keratoconus samples. Thereafter, protein-protein interactions network was generated to identify hub ion channels in network. Furthermore, the pathway map was constructed to depict calcium signalling, MAPK signalling, synthesis and secretion of cortisol, and cAMP signalling. The 19 FDA- approved drugs that interact with the 5 deregulated ion channels were identified.

**Conclusion:**

Down-regulation of voltage-gated calcium channels can be attributed to reduced cell proliferation and differentiation. Additionally, deregulated ion channels in 3’,5’- cyclic adenosine monophosphate signalling may be responsible for elevated cortisol level in progressive keratoconus patients.

## Introduction

Keratoconus is an eye disease that causes bilateral ectasia, progressive thinning, and corneal conical protrusion. Its prevalence is 1 in 2000 people in the general population [1]. Optical aberrations are due to structural changes in the corneal layers, resulting in loss of vision [1]. If stromal thinning and scarring worsen, corneal transplant surgery is required [2]. Keratoconus progression can be affected by environmental factors including frequent eye rubbing or contact lens use, or genetically determined [3]. A number of genetic susceptibility loci have been suggested in keratoconus. Genetic heterogeneity is accountable for the onset and progression of keratoconus [3].

Different types of cells in ocular tissues are essential for maintaining corneal and lens transparency, and proper eye shape [4]. Ion channels are expressed in these cells for housekeeping functions [4]. They are critical in the conversion of light into electrical signals and the regulation of tissue transparency [4]. The majority of these channels have been proposed as potential therapeutic targets [4]. Drugs that activate/block these channels and strategies to modulate their expression, are being developed to treat ocular pathologies [4]. Ion channels expressed by the corneal epithelium and endothelium cells play important roles in ocular pathology [4]. Transient receptor potential cation channels family (TRP) members including TRPV1- 4, TRPM8, TRPA1 and TRPC4 have also been identified in corneal cells [4]. TRPV1 has been identified to be present in the epithelium, stroma, and endothelium of the cornea [5–7]. TRP channel expression has been found to be altered in uveal melanoma [8]. *P2RX7* mRNA expression was found to be higher in corneas of diabetic patients [4]. Potassium channel activity changes have been linked to mitogenic and apoptotic responses induced by growth factors, cytokines, and stress [9]. Fast intracellular potassium loss caused by increased channel activity shrinks epithelial cells, activating several intracellular effectors and triggering death [10–13]. Aquaporins aid to regulate fluid balance and water content in the ocular tissues [4]. Aquaporins present in cornea include AQP1, AQP3, AQP4, and AQP5 [14–19]. Regulation of the water content is necessary for corneal transparency; its variations alter the width and spacing of stromal collagen fibres, which leads to a loss of transparency [4]. A study which found decreased expression levels of *AQP5* in the corneal epithelium of affected patients suggested that *AQP5* loss of function may perform a role in the pathophysiology of keratoconus [20]. Since ion channels have an important role in ocular tissues and their deregulation is known to be involved in ocular pathologies, it is crucial to understand the potential ion channel targets for the better management of keratoconus.

A large number of sequence variants have been identified in keratoconus [21–25]. However, as these variations were specific to family or population, it was not identified as a cause of keratoconus in the general population. Along with DNA-based study, transcriptomics studies can give crucial information to understand keratoconus aetiology [26, 27]. There is no study to the best of our knowledge that describes alterations in ion channels in patients with keratoconus. Here, the transcriptome of patients with keratoconus was analysed for corneal epithelial samples to understand and gain insights into the alterations in the ion channels. Differential gene expression analysis was carried out to obtain quantitative alterations in ion channels expression levels. These expression patterns were further analysed by performing correlation analysis for altered ion channels in keratoconus patients. Furthermore, generation of protein-protein interaction networks resulted in the identification of hub ion channels. Thereafter, pathways associated with altered ion channels were identified in order to obtain potential ion channels as targets in keratoconus. Drugs that interact with potential ion channels were identified. Most likely, ion channels can act as potential drug targets for better management of patients with keratoconus.

## Materials and methods

Supplementary Fig. 1 depicts the overall workflow.

### Data collection

#### Gene list collection

The list of ion channels was downloaded from HGNC database (accessed on 11^th^ March 2022) [28]. Total 374 ion channels were retrieved.

#### Transcriptome sequencing data collection

Three RNA sequencing (RNA-Seq) datasets including GSE151631, GSE112155, GSE77938 were downloaded from Gene Expression Omnibus database and one RNA-Seq dataset with identifier PRJNA799648 was downloaded from BioProject database [29, 30]. The control samples used from these datasets were corneal epithelium of myopic patients with no evidence of corneal damage or chronic pathology and without ocular or other systemic diseases samples [31–34]. The datasets were selected based on the confirmed diagnosis of keratoconus in patient samples and absence of any corneal pathology in the control samples [31–34]. The details of these datasets are provided in the Supplementary Information Tables 1 and 2.

#### Data processing

Quality check was done for raw reads using FastQC, and quality trimming was done using Trimmomatic (v0.39) to obtain the clean reads [35, 36]. In the quality trimming 2 mismatches were allowed in the seed alignment, read score threshold was set to 30, cut-off for the bases at the start and end of a read was set to 3, cut-off for minimum read length was set to 36.

Then these reads were aligned to human genome GRCh38.p13 using STAR RNA-Seq aligner (v2.7.10a) [37]. Further, gene quantification for these aligned reads was done using HTSeq (v1.99.2) [38]. HTSeq output files were merged using R (v4.2.2) script [38].

#### Identification of differentially expressed genes (DEGs)

Differential gene expression analysis of 110 corneal samples (49 control and 61 keratoconus) from four datasets was carried out using DESeq2 (v4.1.2) [39]. The technical replicates in the dataset were considered using collapsedReplicates function in R [39]. The genes with padj < 0.05 and log2 fold change (log2FC) > 1 were chosen as up-regulated and the genes with padj < 0.05 and log2FC < −1 were chosen as down-regulated genes. Deregulated ion channels were parsed from all the deregulated genes using customized Python script.

#### Functional enrichment analysis

Functional analyses of DEGs were performed using Enrichr [40]. Obtained biological processes and cellular components were filtered based on *p*-value ≤0.05.

#### Correlation analysis for expression of differentially expressed ion channels

Customized R (v4.2.2) script was used to plot correlation among deregulated ion channels’ expression for control and keratoconus samples. “goodSamplesGenes” function of Bioconductor package “Weighted gene co-expression network analysis” was used to obtain outlier ion channels so that these ion channels can be excluded from the further analysis [41]. DESeq2 normalised read counts were used for calculating the correlation matrix. Method was set to “pearson” for calculating correlation coefficients. Further, the plots were generated using the “corrplot” function of R [42]. “cor.test” function was used to calculate the significance of the correlations between deregulated ion channels [43].

#### Construction of protein-protein interaction (PPI) networks

PPI networks were built using the STRING (v11.0) database to assess the functional associations among the products of differentially expressed ion channels [44, 45]. FDR stringency was set to 1% and medium confidence score (0.400) was used as the minimum required interaction score. Disconnected nodes in the network were removed. The network was analysed using the Network Analyzer plugin of Cytoscape (v3.9.1) [46]. Additionally PPI networks were generated for all the deregulated genes using stringApp provided in Cytoscape (v3.10.1) [46].

#### Generation of a pathway map

A data mining-based strategy was used to identify the reactions pertaining to deregulated ion channels in patients with keratoconus and the underlying altered pathways. Furthermore, several reactions, including transportation, and catalysis were annotated [47, 48]. Thereafter, a pathway map was constructed in Graphical Pathway Markup Language (GPML) format using PathVisio (v3.3.0) [49]. Entities such as proteins and small molecules are represented by nodes and relationships among the nodes are represented by edges.

#### Drugs interacting with deregulated ion channels

The list of drugs that interact with deregulated ion channels were parsed from the list of drug-gene interactions downloaded from The Drug-Gene Interaction Database [50]. The drugs that interact with ion channels depicted in the pathway map with known mode of interaction were parsed and were represented in the form of heatmap using customized Python (v3.11) script.

## Results

### Differential gene expression analysis

Total 4616 genes were found to be deregulated out of which 531 up-regulated and 4085 down-regulated genes. Furthermore, 75 ion channels were found as deregulated in keratoconus patient samples out of which 12 were up-regulated and 63 were down-regulated. Table 1 represents the top 10 up- and down-regulated ion channels respectively. List of all the deregulated genes is provided in Supplementary Information Table 3 whereas a detailed list of deregulated ion channels is provided in Supplementary Information Table 4.

**Table 1:** Top 10 up- and down- regulated ion channels.

Ion channels	Gene description	Log 2 FC	Deregulation
*GJD2*	Gap junction protein delta 2	2.736438073	Up-regulated
*GJA10*	Gap junction protein alpha 10	1.795833854	Up-regulated
*HTR3C*	5-hydroxytryptamine receptor 3C	1.462856513	Up-regulated
*SCN1A*	Sodium voltage-gated channel alpha subunit 1	1.369409422	Up-regulated
*SCN2A*	Sodium voltage-gated channel alpha subunit 2	1.358126994	Up-regulated
*GABRA4*	Gamma-aminobutyric acid type A receptor alpha 4 subunit	1.331086026	Up-regulated
*KCNB2*	Potassium voltage-gated channel subfamily B member 2	1.269826142	Up-regulated
*GRIK3*	Glutamate ionotropic receptor kainate type subunit 3	1.243676313	Up-regulated
*GLRA4*	Glycine receptor alpha 4	1.241659463	Up-regulated
*RYR2*	Ryanodine receptor 2	1.147361486	Up-regulated
*AQP9*	Aquaporin 9	−5.19249014	Down-regulated
*KCNJ5*	Potassium voltage-gated channel subfamily J member 5	−4.093541531	Down-regulated
*KCNA3*	Potassium voltage-gated channel subfamily A member 3	−3.981440323	Down-regulated
*TRPV2*	Transient receptor potential cation channel subfamily V member 2	−3.69511456	Down-regulated
*KCNK2*	Potassium two pore domain channel subfamily K member 2	−3.633864363	Down-regulated
*GJC1*	Gap junction protein gamma 1	−3.546321386	Down-regulated
*TRPA1*	Transient receptor potential cation channel subfamily A member 1	−3.484108588	Down-regulated
*GABRE*	Gamma-aminobutyric acid type A receptor epsilon subunit	−3.465901798	Down-regulated
*MCOLN2*	Mucolipin 2	−3.372953217	Down-regulated
*KCND2*	Potassium voltage-gated channel subfamily D member 2	−3.169429454	Down-regulated

Top 20 deregulated ion channels included aquaporin, voltage-gated sodium channels, gamma-amino butyric acid receptors, gap junction proteins, voltage-gated potassium channels, and transient receptor potential cation channels.

### Functional enrichment analysis

**Fig. 1:**
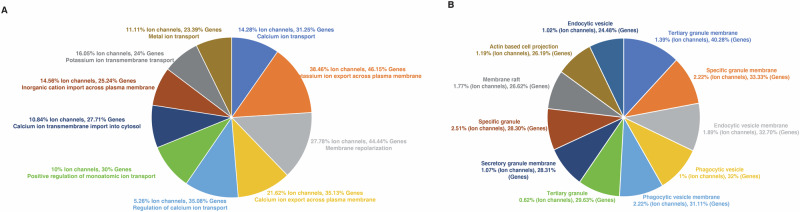
Functional enrichment analysis of differentially expressed genes. **A** Top 10 ranked biological processes enriched for differentially expressed genes including ion channels. **B** Top 10 ranked cellular components enriched for differentially expressed genes including ion channels.

The results were filtered based on involvement of ion channels and *p*-value (≤0.05). Top 10 ranking biological processes and cellular components are represented in Fig. 1. The results obtained indicated that these genes were enriched in biological processes including potassium ion export across plasma membrane, membrane repolarization, calcium ion import across plasma membrane, and cellular components included tertiary granule membrane, specific granule membrane, and endocytic vesicles across plasma membrane.

### Correlation of deregulated ion channels expression

**Fig. 2:**
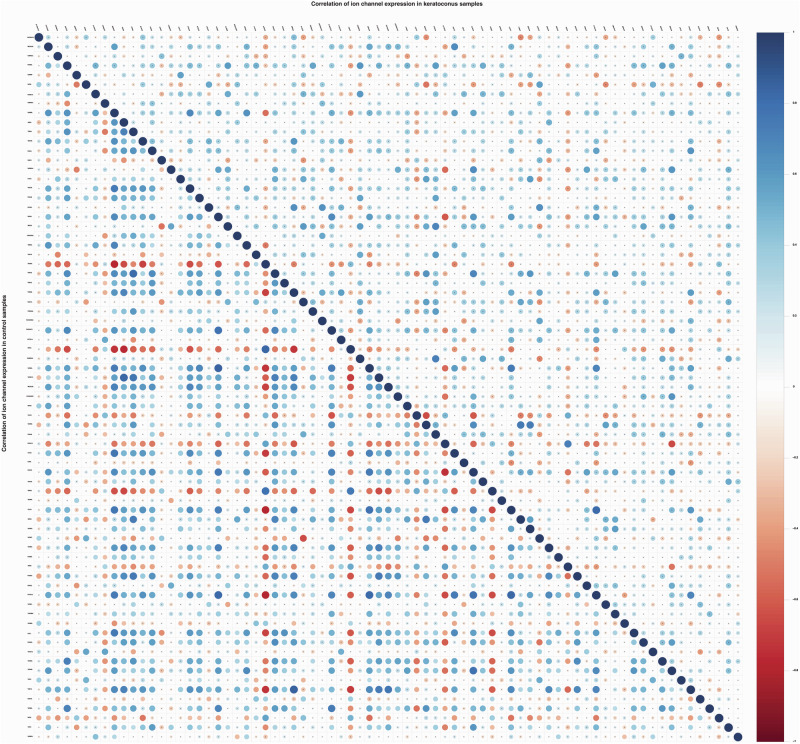
Correlation plot for 75 altered ion channels expression in control and keratoconus patients’ corneal epithelial samples. Lower triangular heatmap on left side represents correlation among 75 ion channels in control samples while upper triangular heatmap on right side represents correlation among deregulated 75 ion channels in keratoconus samples. Negative correlation is represented by brown coloured dots whereas blue coloured dots represent positive correlation. Colour intensity and size of the dots indicate the extent of correlation. No dots represent no correlation.

Further the correlation between deregulated ion channel expression was computed separately for control and keratoconus samples to understand the differences in the correlation between expression of altered ion channels. In the quality check of the expression profile data for outlier detection, none of the outlier ion channels was found in corneal samples. Following this, all the 75 deregulated ion channels were considered for further correlation analysis. Correlation plots along with the significance (*p*-value) are depicted in Fig. 2.

Of all the 2275 correlations, 249 and 113 correlations were found to be significant in control and keratoconus samples respectively. The changes in the correlation (with *p*-value <= 0.05) were observed between *CACNA1C-KCNE4, CACNA1C-KCTD12, CLIC4-KCTD12, CLIC4-TRPV2, GABRE-KCNJ5, GABRE-GRIA3, GABRE-TRPM1, GABRE-SCN2A,GABRE-CLIC6, GABRE-KCNT2, GRIA3-CACNA1C, GRIA3-KCNE4, GRIA3-KCNJ15, GRIA3-KCNT2, GRIA3-CLIC4, GRIA3-KCTD12, GRIA3-KCNQ5, KCNE4-CLIC6, KCNE4-CLIC4, KCNE4-KCTD12, KCNE4-TRPV2, KCNJ15-KCNT2, KCNJ15-ANO6, KCNJ15-KCTD12, KCNK2-KCND2, KCNS2-GRIK1, KCNT2-KCNQ5, KCTD12-TRPV2, MCOLN3-GABRE, TRPC6-KCNS2, TRPC6-KCNT2, TRPM2-CLIC4, TRPM2-KCTD12*, and *TRPM2-TRPV2* and depicted in Supplementary Fig. 2. This change in correlation between ion channels’ expression values in control and keratoconus samples can be indicative of deregulated cellular processes due to altered ion channel expression. Significance of correlation between differentially expressed ion channels is provided in Supplementary Information Table 5.

### Protein-protein interactions networks

PPI network was generated for all the proteins along with the ion channels using STRING (v11.5) database [51]. This PPI network was analysed using Cytoscape (v3.9.1) and visualized using Gephi (v0.9.7) [46] and provided in the Supplementary Fig. 3. PPI network generated using all the differentially expressed genes including ion channels and the modules containing ion channels associated with various cellular processes are provided in Supplementary Fig. 4, respectively.

**Fig. 3:**
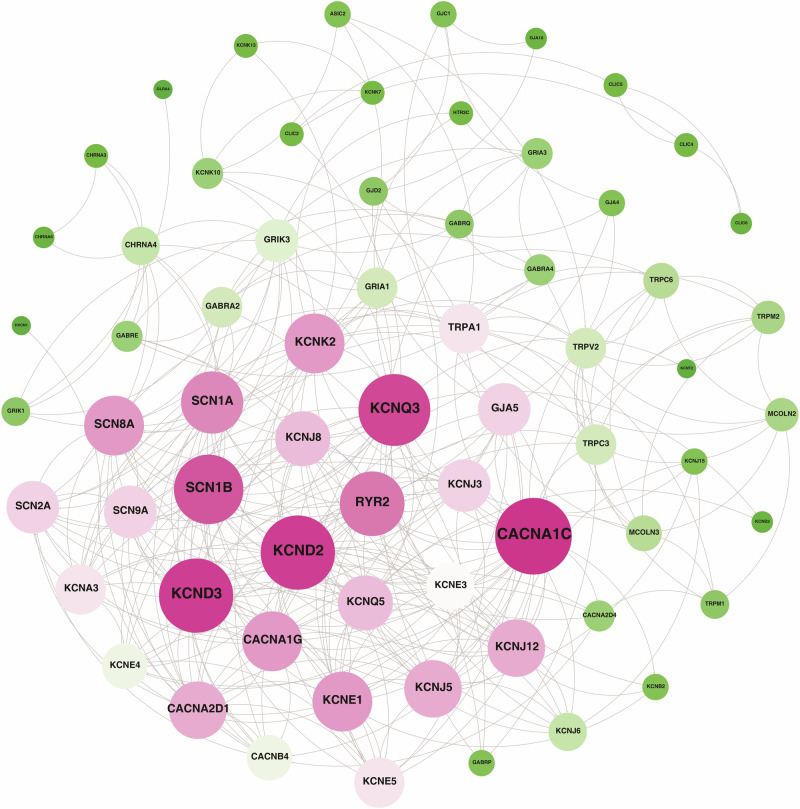
Representation of PPI network for 66 differentially expressed ion channels. Nodes in pink colour represent ion channels with higher degree centrality whereas those with green colour represent ion channels with lesser degree centrality. The colour intensity represents degree centrality of the node.

The PPI network of the 66 of 75 differentially expressed ion channels was constructed. Figure 3 represents a network which contains 66 nodes and 328 edges. Top 20 ranking hub ion channels (Supplementary Information Table 6) were identified based on degree centrality measure (≥15) from the network. These hub ion channels consist of CACNA1C, CACNA1G, KCNK2, RYR2 and CACNA2D1. This indicates that the interaction of ion channels with each other could be altered in keratoconus and that may lead to altered cellular processes involving these ion channels.

### A pathway map of ion channels in keratoconus

**Fig. 4:**
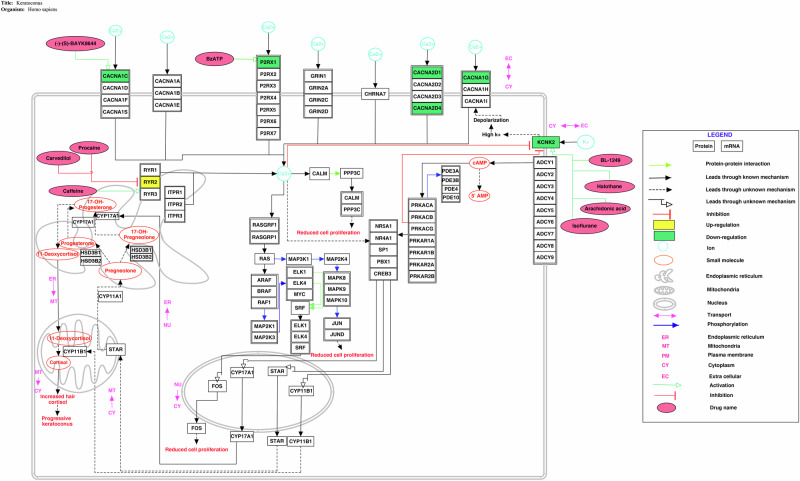
Depiction of pathway reactions that may alter in keratoconus patients due to deregulated ion channels. The pathway map was constructed using PathVisio (v3.3.0). Deregulation of ion channels could be involved in alterations of the cellular processes such as calcium signalling, cAMP signalling, cortisol synthesis and secretion, and MAPK signalling.

Construction of a pathway map that involves deregulated ion channels in various cellular processes can help in comprehending their importance and role in corneal thinning and progression of keratoconus. Figure 4 depicts the deregulated ion channels and underlying cellular processes that are likely to be altered because of the altered ion channels expression in keratoconus. The pathway consists of 130 molecules and 148 reactions. The pathway represents cellular processes such as calcium signalling, cortisol synthesis and secretion, mitogen activated protein kinase (MAPK) signalling, and cAMP signalling that are altered by deregulated ion channels.

Voltage-gated calcium channels were involved in cell proliferation via calcium signalling and MAPK pathway [52, 53]. In the corneal samples of patients with keratoconus, calcium voltage-gated channels including *CACNA1C, CACNA1G, CACNA2D1*, and *CACNA2D4* were found to be down-regulated that could be indicative of reduced cell proliferation as observed in keratoconus [54]. Ion channels involved in cAMP signalling were found to be deregulated that can be attributed to increased cortisol levels in patients with progressive keratoconus [55, 56].

### Drugs interacting with potential ion channels

Of the 75 deregulated ion channels in keratoconus, 55 were found interacting with 625 unique drugs (Supplementary Information Table 7). The pathway map consists of 7 ion channels out of which 5 ion channels were found to interact with 19 FDA approved drugs. Mode of interaction for 19 drugs were known and are represented in Supplementary Fig. 5.

## Discussion

Keratoconus is multifactorial degenerative eye disorder that involves conical protrusion of eyes eventually leading to loss of corneal integrity and vision. A large number of sequence variants have been identified in keratoconus however, it could not be identified as involved in the keratoconus because of their specificity to family or population [21–25]. This implicates the importance of studying transcriptomic data to identify deregulated genes in the keratoconus. Maintaining corneal homeostasis is essential for proper function, requiring the regulation of water balance and optical transparency. Various ion channels are expressed in the cornea, including aquaporins, sodium, chloride, potassium, and calcium channels [57–61].

The current study was focused on identifying deregulated ion channels and the underlying altered processes using computational analysis. Functional enrichment analysis indicated that calcium and potassium ion transports can have an important role in the pathogenesis of keratoconus.

Correlation of ion channels expression was studied to identify changed correlation between ion channel expression in keratoconus patients as compared to the control samples. Out of 75 deregulated ion channels, top 20 ranking hub genes also consisted of some of the ion channels for which correlation was altered including *CACNA1C, KCNJ5, SCN2A, KCNQ5, KCNK2*, and *KCND2*.

This changed correlation between ion channel expression suggested the altered protein-protein interaction and cellular pathways that might be involved in keratoconus. Furthermore, the pathway map was constructed to summarize the potentially altered pathways in keratoconus patients. The pathway map identified deregulation in calcium signalling cAMP signalling, cortisol synthesis and secretion, and MAPK pathway.

### Calcium signalling and MAPK pathway

Calcium ions transported via voltage-gated calcium channels, and purinergic receptors bind to the calcium-binding protein calmodulin, which further binds to and triggers the activity of calcineurin protein phosphatase. Cell proliferation is regulated by the activation of calcineurin and its downstream dephosphorylation activity [62, 63]. An increase in intracellular Ca^2+^ positively regulates Ras signalling leading to increased extracellular signal-reductase kinase (ERK) phosphorylation [64]. RAS guanyl-releasing protein 1 (*RASGRP1*) was found to be down-regulated. This is known to be involved in cellular differentiation proliferation [65]. RASGRP1 gets activated by phosphorylation at threonine present at 184^th^ position and binding of calcium by EF hand motifs [66]. Henceforth, the down-regulated calcium channels *CACNA1C, P2RX1, CACNA2D1, CACNA2D4*, and *CACNA1G* along with *RASGRP1* have high probability of contributing to decreased cell proliferation and differentiation. Functional enrichment analysis results were aligning with this. It included enriched biological processes such as calcium ion export across plasma membrane, calcium ion transport, and calcium ion transmembrane import into cytosol.

The phosphorylated ERK phosphorylates ELK1 and ELK4 that are ETS-domain ternary complex factors and form complex with serum response factor (SRF) that leads to increased transcription of *FOS* gene involved in cell proliferation [52, 67]. N-terminal transactivation domain of c-Jun is phosphorylated by c-Jun N-terminal kinase that promotes DNA binding by promoting the dephosphorylation of sites close to the C-terminal DNA-binding domain and is involved in cell proliferation [67]. Down-regulation of *JUN* could be involved in reduced cell proliferation as it has an important role in regulation of corneal endothelium [68]. Its down-regulation could be attributed to decrease in the cell proliferation. Additionally, c-Jun can facilitate transcriptional activation when L-type voltage-gated calcium channels are activated [69]. This implies that down-regulation of *CACNA1C* (L- type voltage-gated calcium channel) and *JUN* genes could be contributing to reduced cell proliferation.

Additionally, *MYC* gene that was down-regulated is known to be involved in corneal epithelium stratification, differentiation, and surface exfoliation [70]. Its activity depends on its phosphorylation [70]. Ca^2+^ signalling triggers intracellular biochemical responses by activating various calcium-binding proteins including calcineurin [71]. Intracellular Ca^2+^ concentration modulated stability of *MYC* [71]. Calcineurin which is a Ca^2+^-dependent protein phosphatase positively regulates c-Myc expression [71]. This suggests that the down-regulated calcium channels and *MYC* could be involved in reduced cell proliferation

As a consequence, down-regulated voltage-gated calcium channels in *CACNA1C, CACNA1G, CACNA1D1, and CACNA2D4*, and purinergic receptors *P2RX1* along with the down- regulated transcription factors including *JUN* and *MYC* can be attributed to reduced cell proliferation in keratoconus.

### Cortisol synthesis and secretion

Protein kinase A is activated by cAMP, which further activates and induces the proteins and steroidogenic enzymes that convert cholesterol to cortisol [72–75].

Steroidogenesis enzyme genes may play a role in sex hormone metabolism and the risk of high-myopia [76]. Myopia is one of the symptoms observed in keratoconus patients [77]. 17 alpha-hydroxylase encoded by *CYP17A1* and 3 beta-hydroxysteroid dehydrogenases encoded by *HSD3B1* are involved in the biogenesis of progesterone from pregnenolone [76]. It has been observed that *NR4A1* is down-regulated. It has been suggested that reduced expression of the steroid-retinoid hormone receptor encoded by *NR4A1*, is known to be involved in apoptosis and may also be part of a deregulated injury response in keratoconus [32]. Moreover, *SP1* was found to be up-regulated in keratoconus [78]. Here it could be hypothesized that deregulation of Ca^2+^ channels could be attributed to deregulation of the *SP1* that could be involved in increased cortisol synthesis and secretion. Additionally, disorganization of corneal collagen matrix and loss of corneal barrier function was observed in *PBX1* knocked-out mice [79]. Deregulation of calcium ion channels could be involved in deregulated influx of Ca^2+^ ions causing dysfunction of *PBX1* and increased cortisol synthesis and secretion.

cAMP-mediated inhibition of KCNK2 by a mechanism that is dependent on Ca^2+^ entry through T-type and L-type Ca^2+^ channels functions synchronically with cAMP to increase cortisol production [72]. The potassium channel *KCNK2* was found to be down-regulated with log2FC −3.633864363 indicating that deregulation of this ion channel might have high probability of contributing to increased cortisol levels in patients with progressive keratoconus[55, 56].

Ion channels are the second largest class of membrane proteins that are considered as drug targets. Presence of these channels on membranes makes them potential drug targets. A dihydropyridine compound, BAY K8644 is the activator of CACNA1C [50]. It enhances the calcium channel activity by increasing the open time and the single channel conductance. Benzoyl ester of ATP (BzATP) can activate P2X1 [80]. In vitro study showed that BzATP can elevate cell proliferation [80]. It has been reported that BzATP can enhance corneal wound healing outcomes [81]. Halothane and Isoflurane were found to activate KCNK2 in HEK-293 cells [82]. Arachidonic acid is also known to increase the activity of KNCK2 [83]. A compound from the fenamate class of nonsteroidal anti-inflammatory drugs, BL-1249, is the activator of KNCK2 channel [50]. Procaine acts as a blocker of the channel RYR2 [84]. Additionally, carvedilol is an antagonist for the same intracellular channel RYR2 [85].

The altered ion channels and their interactions with the drugs need to be studied in vitro and in vivo to target potential ion channels and their associated pathways.

## Conclusions

Differentially expressed ion channels were determined using transcriptomic data of patients with keratoconus. Potentially altered pathways including calcium signalling, cAMP signalling, and cortisol synthesis and secretion were identified. Additionally, identification of drugs interacting with the deregulated ion channels could be helpful in drug repurposing where further experimental validations will be required. Overall, this computational approach of studying deregulation of ion channels in keratoconus will be helpful in better management of patients with keratoconus.

## Summary

### What was known before

- To the best of our knowledge molecular alterations in ion channels have not been reported in patients with keratoconus.

### What this study adds

- 75 Differentially expressed ion channels were identified using transcriptomic data of patients with keratoconus
- Potentially altered pathways including calcium signalling, cAMP signalling, and cortisol synthesis and secretion were identified
- Additionally, 19 FDA-approved drugs interacting with the 5 deregulated ion channels were identified.

## Supplementary information

Supplementary File 1 Legends for supplementary information.

Supplementary File 2 Workflow for the identification of altered ion channels.

Supplementary File 3 Comparison of correlation of ion channels expression for control and keratoconus.

Supplementary File 4 Protein-protein interaction network of differentially expressed genes.

Supplementary File 5 Highly connected gene modules that included ion channels which are involved in cellular processes of the pathway map.

Supplementary File 6 Depiction of interactions of drugs with deregulated ion channels.

Supplementary File 7 Sample details with and without replicates.

Supplementary File 8 List of 531 up and 4085 down-regulated genes in keratoconus.

Supplementary File 9 List of deregulated ion channels in keratoconus.

Supplementary File 10 Significant correlations between ion channel expression and their respective p-values.

Supplementary File 11 Hub genes predicted from the PPI network of deregulated ion channels

Supplementary File 12 List of ion channels interacting with drugs.

## Data Availability

Publicly available datasets were analysed in this study. The data can be accessed using the following resources: GEO DataSets database (https://www.ncbi.nlm.nih.gov/gds/), BioProject database (https://www.ncbi.nlm.nih.gov/bioproject/) accessed on 25 September 2022. Customized R scripts used for identification of differentially expressed genes and keratoconus ion channel pathway reaction data are available in the GPML format through the GitHub repository via the following URLs: (https://github.com/js-iob/Keratoconnus_ICs/blob/main/DESeq2_allDatasets_collapseDup.R, https://github.com/js-iob/Keratoconnus_ICs/blob/main/75_IC_pathway_121523.gpml) accessed on 15 Dec 2023. Cutomized R script for correlation analysis of differentially expressed ion channels is available through the GitHub repository via following URL (https://github.com/js-iob/Keratoconnus_ICs/blob/main/correlation_collapsedReplicates_090123.R) last accessed on 15 Dec 2023.
