# Correction: Computational approaches for identifications of altered ion channels in keratoconus

**DOI:** 10.1038/s41433-024-03493-4

**Published:** 2025-01-02

**Authors:** Kiran Bharat Gaikwad, Jayavigneeswari Suresh Babu, K. T. Shreya Parthasarathi, Janakiraman Narayanan, Prema Padmanabhan, Akhilesh Pandey, Seetaramanjaneyulu Gundimeda, Sailaja V. Elchuri, Jyoti Sharma

**Affiliations:** 1 Manipal Academy of Higher Education, Manipal, Karnataka 576104 India; 2 Institute of Bioinformatics, International Technology Park, Bangalore, 560066 India; 3 Department of Nanobiotechnology, Vision Research Foundation, Sankara Nethralaya Campus, Chennai, 600006 India; 4 Department of Cornea, Medical Research Foundation, Sankara Nethralaya, Chennai, 600006 India; 5 Department of Laboratory Medicine and Pathology, Mayo Clinic, Rochester, MN 55905 USA; 6 Center for Individualized Medicine, Mayo Clinic, Rochester, MN 55905 USA

**Keywords:** Corneal diseases, Drug discovery

Correction to: *Eye* 10.1038/s41433-024-03395-5, published online 17 October 2024

In the abstract, the following sentence “The 19 FDA-approved drugs that interact with the 6 deregulated ion channels were identified” was corrected to read “The 19 FDA-approved drugs that interact with the 5 deregulated ion channels were identified.” The original article has been corrected.

