# Supplementary material for: Computational approaches for identifications of altered ion channels in keratoconus
Source: Eye (Lond). 2024 Oct 17;39(1):145–53. doi: 10.1038/s41433-024-03395-5 (PMC11733014; doi:10.1038/s41433-024-03395-5)
Supplement: Supplementary File 1 — Legends for supplementary information. [file 41433_2024_3395_MOESM1_ESM.docx]

**Computational Approaches for Identifications of Altered Ion Channels in Keratoconus**

**Kiran Bharat Gaikwad^1,2^, Jayavigneeswari Suresh Babu^3^, K.T. Shreya Parthasarathi^1,2^, Jankiraman Narayanan^3^, Prema Padmanabhan^4^, Akhilesh Pandey^5,6^, Seetaramanjaneyulu Gundimeda^1,2^, Sailaja V. Elchuri^3*^ and Jyoti Sharma^1,2*^**

^1^ Manipal Academy of Higher Education, Manipal, Karnataka 576104, India

^2^ Institute of Bioinformatics, International Technology Park, Bangalore 560066, India

^3^Department of Nanobiotechnology, Vision Research Foundation, Sankara Nethralaya Campus, Chennai, India.

^4^ Department of Cornea, Medical Research Foundation, Sankara Nethralaya, Chennai, India.

^5^ Department of Laboratory Medicine and Pathology, Mayo Clinic, Rochester, MN 55905, USA;

^6^ Center for Individualized Medicine, Mayo Clinic, Rochester, MN 55905, USA

***Correspondence:**

Dr. Sailaja V. Elchuri, Department of Nanobiotechnology, Vision Research Foundation, Sankara Nethralaya Campus, Chennai, India

sailaja.elchuri@gmail.com

Dr. Jyoti Sharma, Institute of Bioinformatics, International Technology Park, Bangalore 560066, India

jyoti@ibioinformatics.org

**Supplementary Information**

**Supplementary Figure 1:** Workflow for the identification of altered ion channels, associated pathways, and drugs interacting with deregulated ion channels in keratoconus.

**Supplementary Figure 2:** Comparison of correlation of ion channels expression for control and keratoconus. Blue and Orange lines represents correlation values between ion channels provided on X-axis for control and keratoconus respectively

**Supplementary Figure 3:** Protein-protein interaction network of differentially expressed genes.

**Supplementary Figure 4:** Highly connected gene modules that included ion channels which are involved in cellular processes of the pathway map.

**Supplementary Figure 5:** Depiction of interactions of drugs with deregulated ion channels that are involved in alterations of the cellular processes.

**Supplementary Information Table 1 and 2:** Sample details with and without replicates

**Supporting Information Table 3:** List of 531 up and 4085 down-regulated genes in keratoconus

**Supporting Information Table 4:** List of deregulated ion channels in keratoconus

**Supplementary Information Table 5:** Significant correlations between ion channel expression and their respective p-values

**Supporting Information Table 6:** Hub genes predicted from the PPI network of deregulated ion channels

**Supporting Information Table 7:** List of ion channels interacting with drugs
