# Supplementary material for: Computational approaches for identifications of altered ion channels in keratoconus
Source: Eye (Lond). 2024 Oct 17;39(1):145–53. doi: 10.1038/s41433-024-03395-5 (PMC11733014; doi:10.1038/s41433-024-03395-5)
Supplement: Supplementary File 2 — Workflow for the identification of altered ion channels. [file 41433_2024_3395_MOESM2_ESM.docx]

Supplementary Material

**Computational Approaches for Identifications of Altered Ion Channels in Keratoconus**

**Kiran Bharat Gaikwad^1,2^, Jayavigneeswari Suresh Babu^3^, K.T. Shreya Parthasarathi^1,2^, Jankiraman Narayanan^3^, Prema Padmanabhan^4^, Akhilesh Pandey^5,6^, Seetaramanjaneyulu Gundimeda^1,2^, Sailaja V. Elchuri^3*^ and Jyoti Sharma^1,2*^**

**Correspondence:** Jyoti Sharma [jyoti@ibioinformatics.org](mailto:jyoti@ibioinformatics.org), Sailaja V Elchuri [sailaja.elchuri@gmail.com](mailto:sailaja.elchuri@gmail.com)

**Supplementary Information Table 1 Sample details without replicates**

| **BioProject/GEO IDs** | **Number of control samples** | **Number of patient samples** |
| --- | --- | --- |
| PRJNA799648 | 7 | 7 |
| GSE151631 | 7 | 7 (African Americans), 12 (Middle Eastern) |
| GSE112155 | 10 | 10 |
| GSE77938 | 25 | 25 |
| Total | 49 | 61 |

**Supplementary Information Table 2 Sample details with replicates**

| **BioProject/GEO IDs** | **Number of control samples** | **Number of patient samples** |
| --- | --- | --- |
| PRJNA799648 | 7 | 7 |
| GSE151631 | 7 | 7 (African Americans), 12 (Middle Eastern) |
| GSE112155 | 10 | 10 |
| GSE77938 | 60 | 68 |
| Total | 84 | 104 |
