# Supplementary figures and images for: Computational approaches for identifications of altered ion channels in keratoconus
Source: Eye (Lond). 2024 Oct 17;39(1):145–53. doi: 10.1038/s41433-024-03395-5 (PMC11733014; doi:10.1038/s41433-024-03395-5)

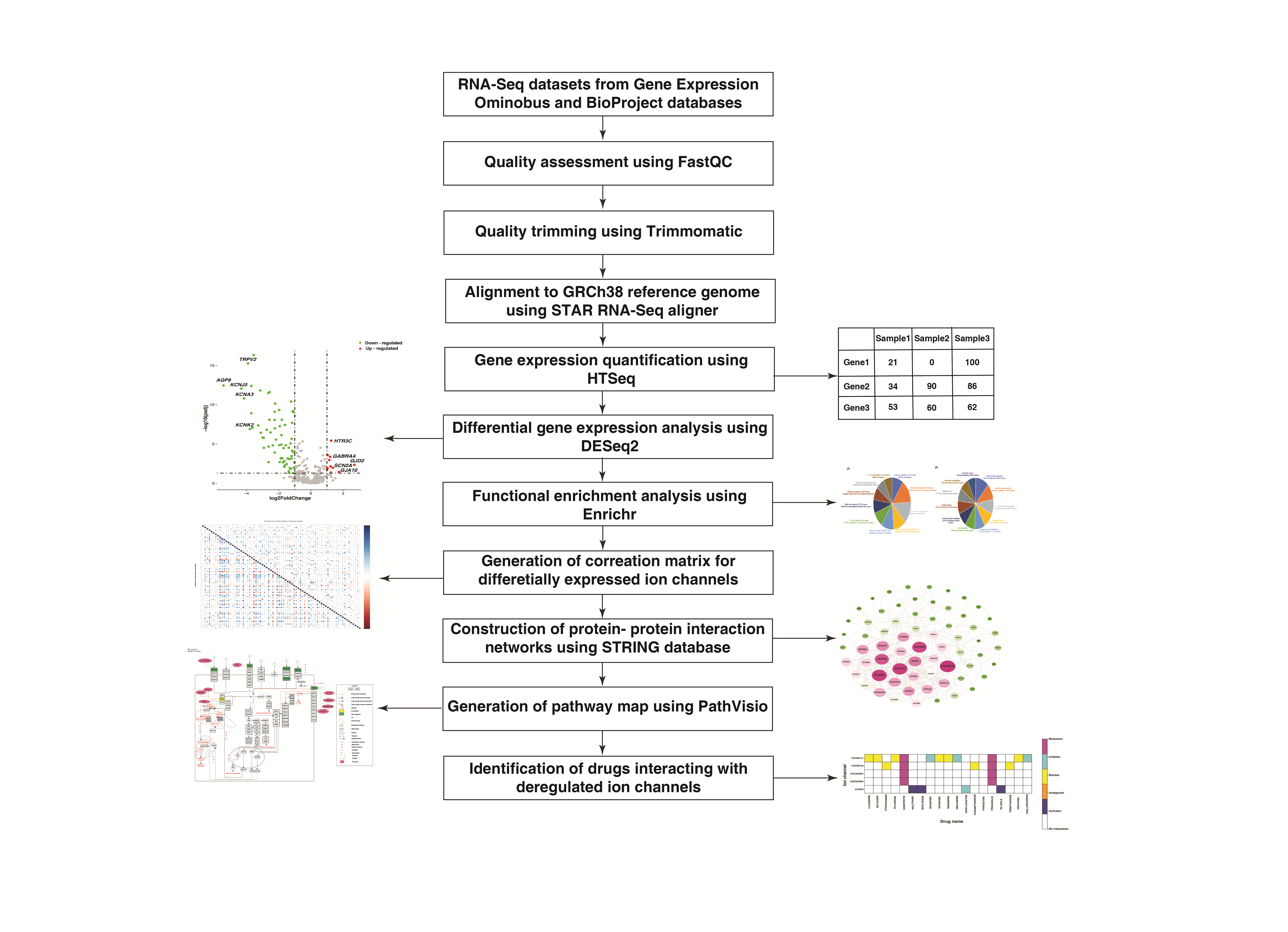

Supplement: Supplementary File 8 — List of 531 up and 4085 down-regulated genes in keratoconus. [file 41433_2024_3395_MOESM8_ESM.tif]

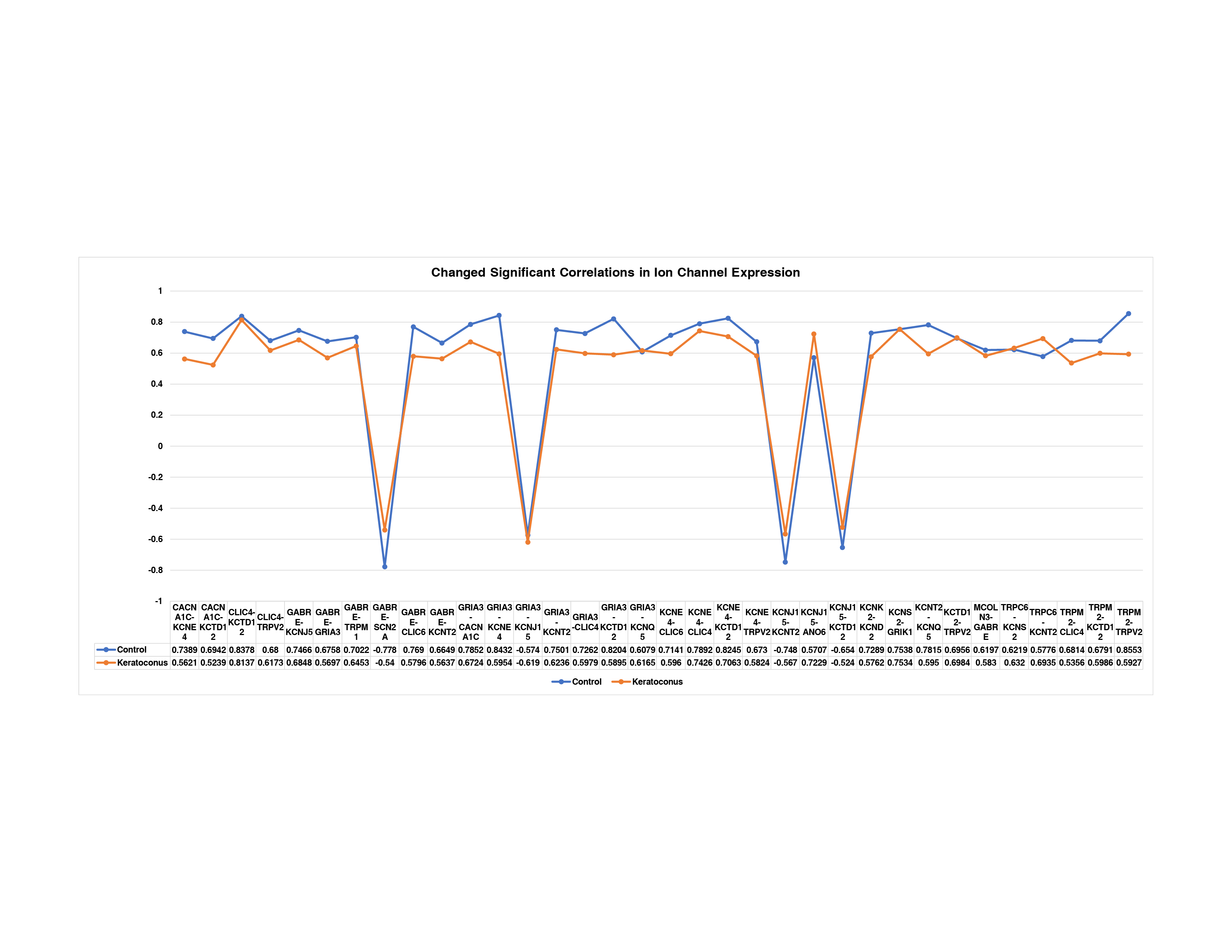

Supplement: Supplementary File 9 — List of deregulated ion channels in keratoconus. [file 41433_2024_3395_MOESM9_ESM.tif]

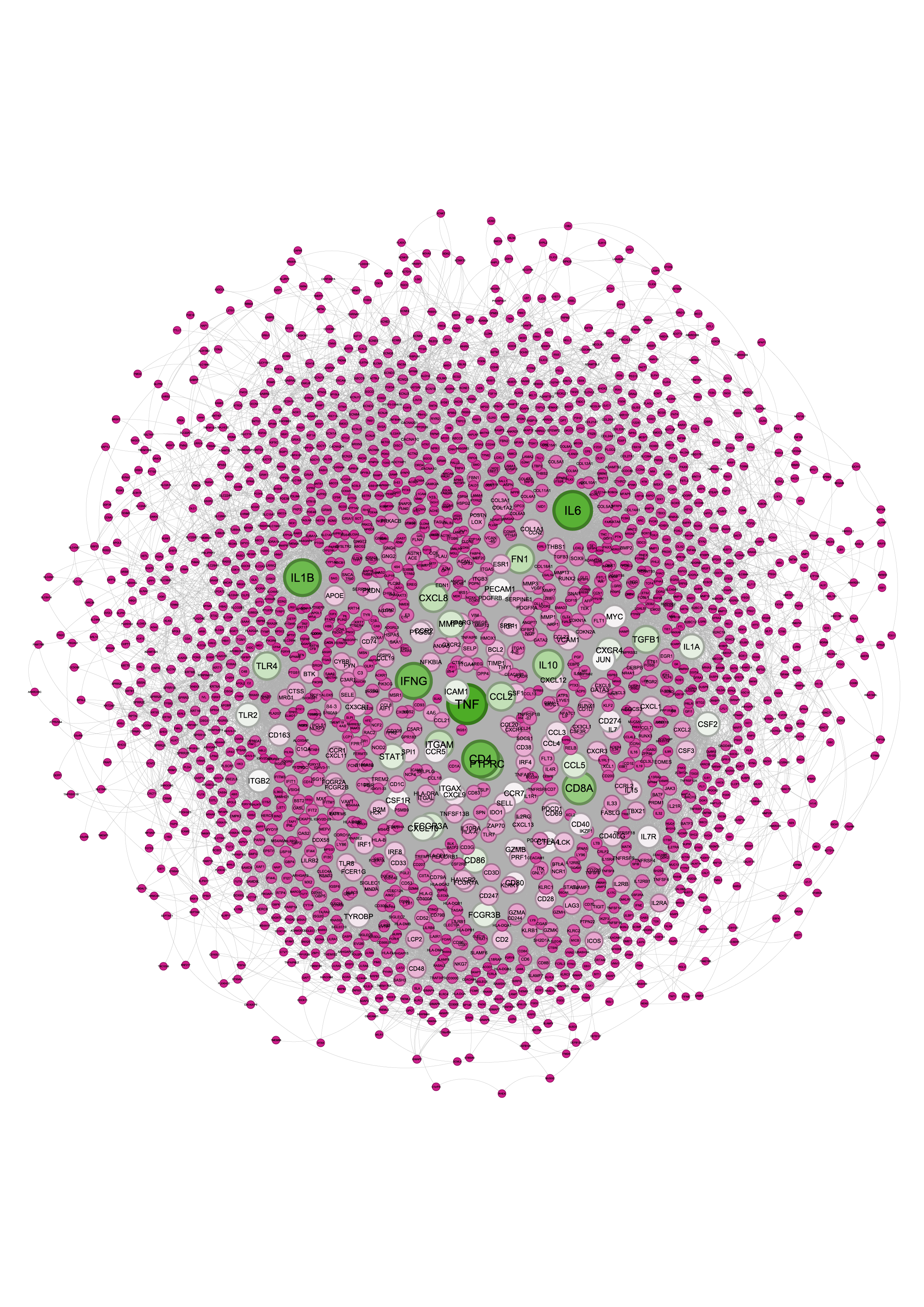

Supplement: Supplementary File 10 — Significant correlations between ion channel expression and their respective p-values. [file 41433_2024_3395_MOESM10_ESM.tif]

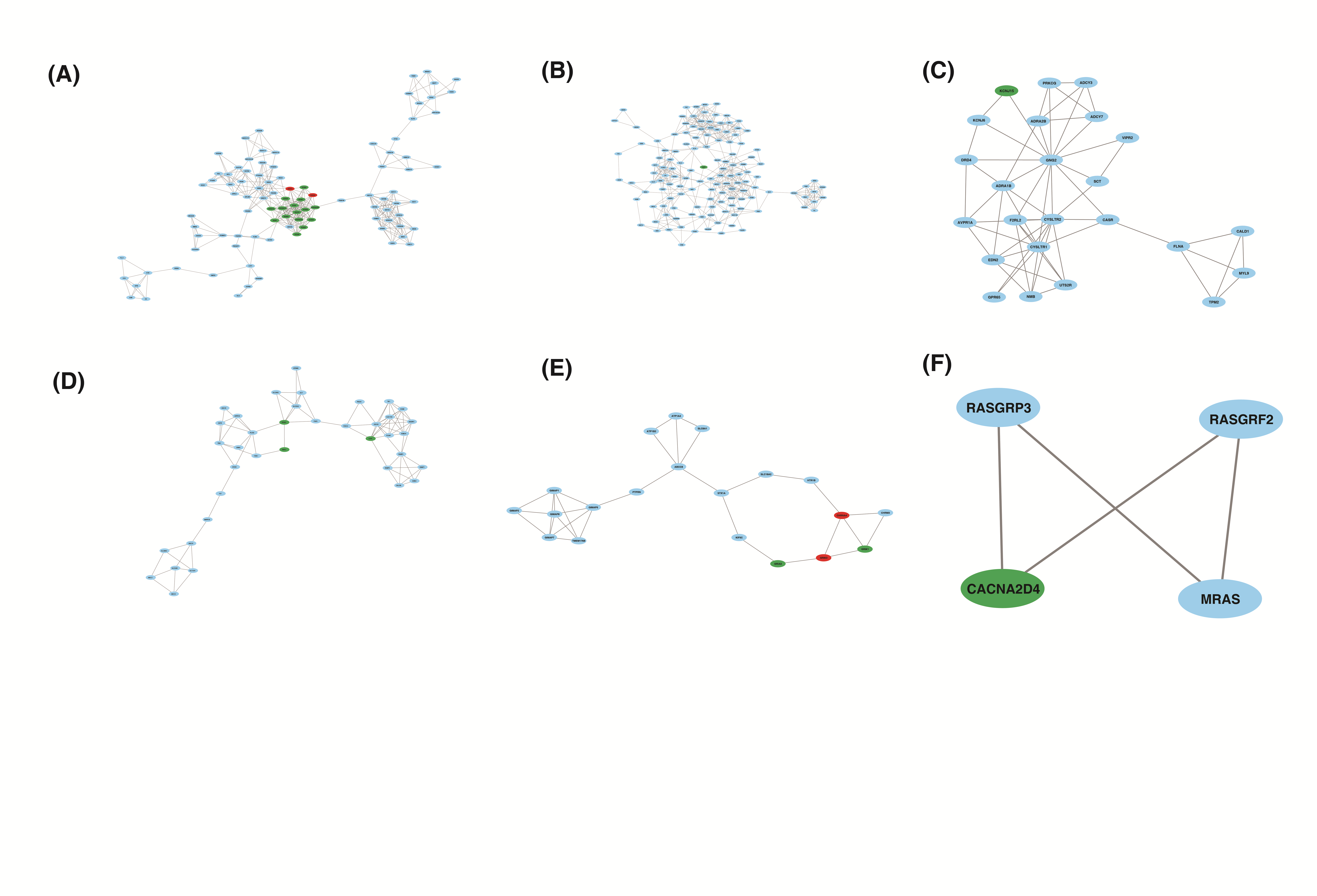

Supplement: Supplementary File 11 — Hub genes predicted from the PPI network of deregulated ion channels [file 41433_2024_3395_MOESM11_ESM.tif]

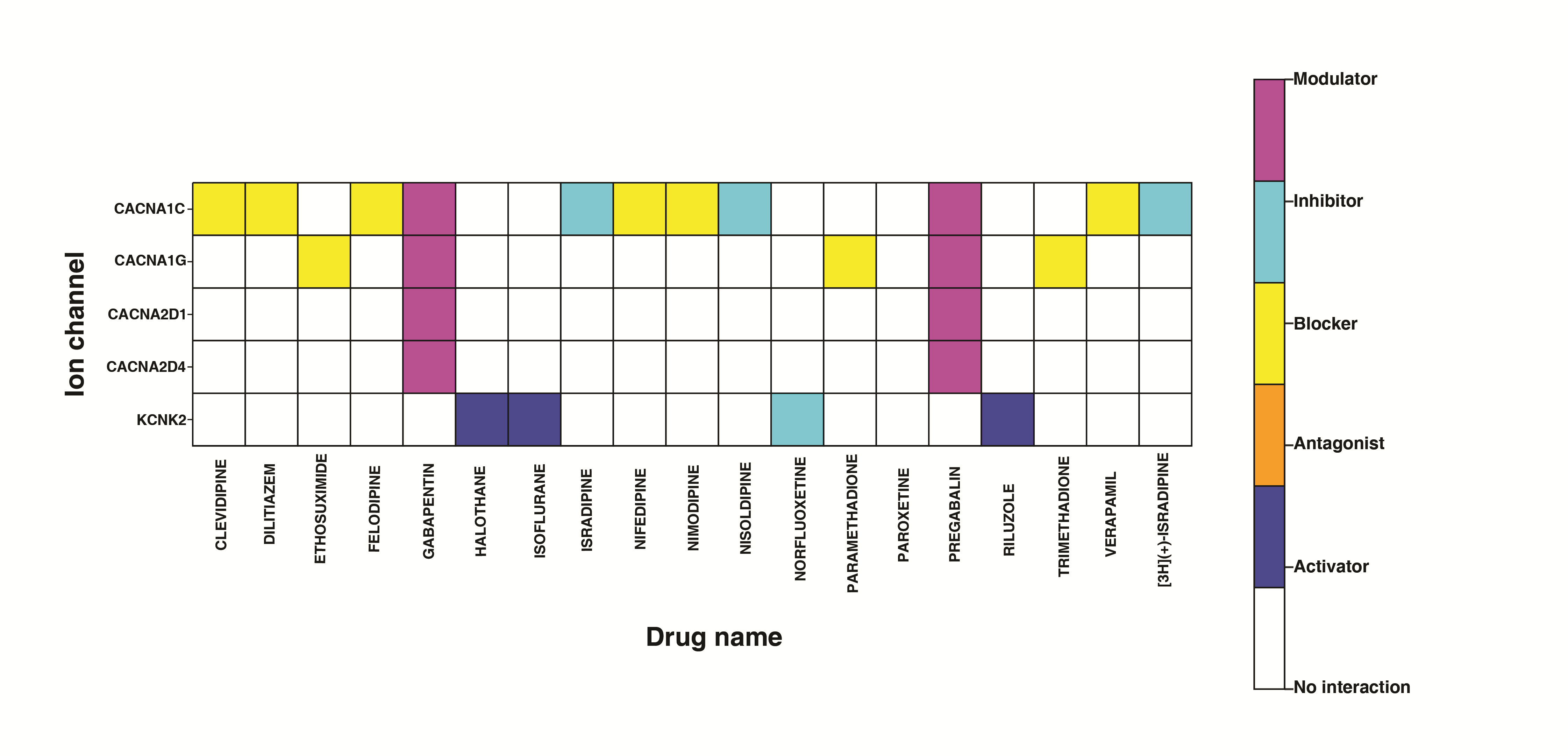

Supplement: Supplementary File 12 — List of ion channels interacting with drugs. [file 41433_2024_3395_MOESM12_ESM.tif]
